# Let the question determine the methods: descriptive epidemiology done right

**DOI:** 10.1038/s41416-020-1019-z

**Published:** 2020-08-20

**Authors:** Sara Conroy, Eleanor J. Murray

**Affiliations:** 1 Center for Biostatistics, Department of Biomedical Informatics, The Ohio State University, Columbus, OH USA; 2 Department of Epidemiology, Boston University School of Public Health, Boston, MA USA

**Keywords:** Research management, Education

## Abstract

What does it mean to control for confounding, and when do we actually need to do it? To answer this, we need a well-defined research question, driven by the goal of the study. For descriptive goals, we explain that confounding adjustment is often not just unnecessary but can be harmful.

## Main

In 2017, Pedersen et al. published a study that explored the potential statistical association of frozen-shoulder and cancer diagnoses.^1^ Recently, discussion of this paper on Twitter criticised the authors for not controlling for confounding.^2–4^ But, what does it mean to control for confounding, and when do we actually need to do it? Confounding is typically defined as a variable that is related to both the main variable of interest and the outcome, but is not on the causal pathway. A directed acyclic graph is a useful tool to help determine if a variable is a confounder.^5^ The decision to control for a confounder depends on the specific scientific question, and typically occurs when the focus of the research question is to investigate a causal relationship between the main variable of interest and the outcome. However, not all research requires a causal question.^6^ Research studies that focus on describing a population of interest are essential building blocks for both causal and predictive frameworks, and do not typically require control for additional variables. To understand the purpose of a study (i.e., descriptive, causal or predictive), it is vital that the goals of the research be clearly explained.^7^ The Pedersen article is a great example of a study with a research question that is not causal and therefore does *not* need to control for confounding. Here, we discuss why the authors were correct not to control for confounding, and how the research question should guide the methods, especially when conducting descriptive epidemiology studies.

### What was the research question?

Pedersen et al.^1^ were motivated by the observation that people who have cancer often develop frozen shoulder, but also that certain types of cancer may be misdiagnosed as frozen shoulder. With this ambiguity, the authors designed a study to address the question: “Is frozen shoulder a warning sign that can identify a group of people who might be at high risk for cancer?” This is not a causal question as the authors are not suggesting that frozen shoulder causes cancer, or that cancer diagnosis causes frozen shoulder.

Two main goals helped answer the research question. First, the authors wanted to describe the incidence of cancer among people with frozen shoulder. Second, they wanted to compare and contrast the incidence of frozen shoulder in the data with the general population (i.e., people who may one day get a cancer diagnosis).

### What is the ultimate goal?

Ultimately, the researchers hope to improve early diagnosis of cancer. Cancer screening is the action/decision that the authors were trying to inform. After describing the population of interest and comparing with other relevant populations, if a difference is observed, there are multiple directions the next study could go. One could build a predictive model of cancer using frozen shoulder information or test a cancer-screening programme that focuses on shoulder patients compared with usual screening patients. These studies could help determine if a larger proportion of cancer patients could be found earlier. The main insight of those next steps is that any hypothetical trial building on the current study would not intervene on shoulder problems but instead on cancer screening.

### But, what about confounding?

Discussion of this paper on Twitter has faulted the authors for not controlling for confounding.^3,4^ However, we argue here that the authors were in fact correct to not control for confounding because confounding is precisely what they hoped to identify. The authors are attempting to find a statistical association between frozen shoulder and cancer. Any statistical relationship is expected to be influenced by other variables (e.g., preclinical cancer). Adjusting for confounding variables in this analysis runs the risk of getting the wrong answer as it might accidentally open a collider path and create an association that is not normally there.^8^ An open collider path that could make it seem like frozen shoulder is a good early warning tool for cancer diagnosis when it actually is not.

The data set covered the entire Danish population, and there was no evidence to screen for cancer based on frozen shoulder. Generalisability would depend on the underlying cancer and frozen-shoulder incidence in other populations (outside of Denmark). For example, if the incidence of cancer was the same, but frozen-shoulder cases were higher in Canada, the association of frozen shoulder and cancer may be smaller. If the incidence of cancer was the same, but frozen-shoulder cases were lower in Canada, the association of frozen shoulder and cancer might be higher compared with this population in this study. In a similar fashion, if both the common cause of frozen shoulder and cancer were higher in Canada, the association between frozen shoulder and cancer would probably be larger, and screening may be warranted.

### The right methods for the right question

The authors could have also asked: “Is frozen shoulder predictive of a high risk of cancer above and beyond other known cancer risk factors?” This question would imply the use of frozen shoulder as a proxy for unknown causes of cancer, and in that case, control for known causes of cancer, or known predictors of cancer could be appropriate. Those other known predictors need not also be causes of frozen shoulder, and are thus not necessarily confounders.^8^

Finally, in keeping with current recommendations from the American Statistical Association and others,^9^ the authors of this paper do not rely solely on *P* values for interpreting their findings. Instead, they consider the magnitude of the association, and conclude that, although statistically significant, the detected association is not big enough to warrant stratified screening in Denmark.^10^

In summary, the paper by Pedersen et al.^1^ is an excellent example of descriptive epidemiology done right. We commend the authors for their clarity in explaining that they are not estimating causal effects, in the clear lack of causal language, and in the inclusion of specific discussion of whether the results suggest that stratified screening programmes could be a useful next step. We recommend this paper to those who teach descriptive epidemiology for use with their students.

## Data Availability

Not applicable.
